# Vitexin Regulates Heat Shock Protein Expression by Modulating ROS Levels Thereby Protecting against Heat-Stress-Induced Apoptosis

**DOI:** 10.3390/molecules28227639

**Published:** 2023-11-17

**Authors:** Tong Wu, Yanan Sheng, Yu Tian, Changyuan Wang

**Affiliations:** 1 College of Food, Heilongjiang Bayi Agricultural University, Xinfeng Road 5, Daqing 163319, China; wutong@byau.edu.cn (T.W.); syn0307@yeah.net (Y.S.); tianyu19971001@byau.edu.cn (Y.T.); 2 National Coarse Cereals Engineering Research Center, Daqing 163319, China

**Keywords:** vitexin, heat stress, ROS, heat shock protein, apoptosis

## Abstract

Heat stress due to high temperatures can cause heat stroke, pyrexia, heat cramps, heart disease, and respiratory diseases, which seriously affect human health. Vitexin has been shown to alleviate heat stress; however, its mechanism of action remains unclear. Therefore, in this study, we used Caco-2 cells to establish a heat stress model and vitamin C as a positive control to investigate the regulatory effects of vitexin on heat-stress-induced apoptosis and the related mechanisms using Cell Counting Kit-8, flow cytometry, real-time quantitative polymerase chain reaction, and Western blot. The results showed that the mRNA expressions of Hsp27, Hsp70, and Hsp90 induced by heat stress could be effectively inhibited at vitexin concentrations as low as 30 μM. After heat stress prevention and heat stress amelioration in model cells based on this concentration, intracellular reactive oxygen species (ROS) levels and the mRNA level and the protein expression of heat shock proteins (Hsp70 and Hsp90) and apoptotic proteins were reduced. In addition, compared with the heat stress amelioration group, the expression of *BCL2* mRNA and its protein (anti-apoptotic protein Bcl-2) increased in the heat stress prevention group, while the expression of *BAX*, *CYCS*, *CASP3*, and *PARP1* mRNAs and their proteins (apoptotic proteins Bax, Cytochrome C, cle-Caspase-3, and cle-PARP1) were decreased. In summary, the heat-stress-preventive effect of vitexin was slightly better than its heat-stress-ameliorating effect, and its mechanism may be through the inhibition of intracellular ROS levels and thus the modulation of the expressions of Hsp70 and Hsp90, which in turn protects against heat-stress-induced apoptosis. This study provides a theoretical basis for the prevention and amelioration of heat stress using vitexin.

## 1. Introduction

Heat stress is the sum of nonspecific response mechanisms that occur in humans or animals when subjected to high-temperature stimuli that they are unable to regulate [1]. Deterioration of the natural environment is expected to lead to a continuous increase in heat stress situations [2,3,4]. Heat stress is a major public health challenge and has seriously affected human health. In daily life, prolonged exposure of the human body to high temperatures may lead to heat stroke, pyrexia, heat cramps, and other conditions [5]. In addition, heat exposure can cause illnesses such as heart, respiratory, and kidney diseases, which can lead to death if not treated in time [6]. Consequently, many countries have developed public health measures for heat wave warnings in response to heat stress. However, these measures are only moderately effective in preventing heat stress [7]. In general, common treatments include rapid cooling and the consumption of fluids and medications [8], but the measures to alleviate heat stress are currently limited, and there is still a lack of effective medications to alleviate heat stress. It is therefore crucial to find effective natural ingredients for the prevention and alleviation of heat stress.

Polyphenols have strong antioxidant properties and can be used as supplements to prevent heat stress [9]. Studies have shown that natural polyphenols, including tea polyphenols, resveratrol, ferulic acid, and curcumin, can alleviate heat stress by decreasing the activity of antioxidant enzymes in the body or by modulating related signaling pathways. Curcumin can alleviate heat-stress-induced oxidative damage by activating the MAPK-Nrf2 signaling pathway through the upregulation of Jnk, Erk, p38, and Nrf2; tea polyphenols alleviate heat stress injury by increasing the activities of antioxidant enzymes (GSH-Px, SOD, and T-AOC) and upregulating the expression of heat shock proteins (Hsp27 and Hsp70); ferulic acid can not only inhibit heat-stress-induced intestinal oxidative stress by decreasing the expression of ROS, MDA, and NO and increasing SOD activity, but also it protects against heat-stress-induced intestinal epithelial barrier dysfunction by increasing the protein expression levels of Akt, Nrf2, and HO-1; dietary resveratrol supplementation can improve the intestinal mucosal morphology and downregulate the mRNA expression of *HSPA1A*, *HSP90AB1*, and *NF*-*κB*. It can also increase the expression of epidermal growth factor in chickens on the 6th, 10th, and 15th day of heat stress, thereby reducing stress-induced intestinal mucosal damage [10,11,12,13,14]. As a natural polyphenol, vitexin, which is present in high concentrations in mung beans, hawthorn, and other foods, has a strong antioxidant activity [15] and has been widely used for the prevention and treatment of various diseases because of its functional and pharmacological activities [16]. It was shown that vitexin could alleviate heat-stress-induced apoptosis by upregulating antioxidant enzyme activities and endoplasmic reticulum-stress-mediated autophagy [17]; however, whether vitexin can relieve apoptosis induced by heat stress by regulating the ROS level and the heat shock protein expression has not been investigated. A previous study by our group found that vitexin could alleviate heat stress to a certain extent in Caco-2 and Mode-k cells, and it was speculated that the antioxidant property of vitexin plays a key role in regulating heat stress [18]; however, to the best of our knowledge, the mechanism related to the modulation of heat stress by vitexin has not been previously explored. In addition, other relevant studies have confirmed that vitexin intervention alleviates high temperature-induced inflammation [19], which is mainly aimed at improving the health and productivity challenges of heat-sensitive dairy cows in the tropics. In this study, we used an in vitro cellular model to simulate the temperature and time of human heat stroke to explore the possible mechanisms of vitexin in preventing and ameliorating the possible effects of heat stress on human intestinal tract.

When an organism is exposed to high temperatures for a long period, its intestinal function is mainly affected, and an impaired intestinal function leads to damage to other organs [20]. Therefore, it is of practical significance to study the mechanism of action of heat stress on the intestinal tract. In recent years, cellular models have been gradually applied to the study of heat stress, and significant research progress has been made in this field [21]. Caco-2 cells are a human intestinal epithelial cell model that functionally and structurally resemble mature small intestinal epithelial cells. These cells are often used as a heat stress model for intestinal epithelial cells because of their mature and stable function [22,23]; therefore, Caco-2 cells were chosen in this study to explore the mechanism of action of vitexin in regulating heat stress.

Apoptosis is the process of programmed cell death [24], which plays an important role in maintaining the growth and development of organisms [25]. Reactive oxygen species (ROS) play a key role in cell signaling and homeostasis in vivo, and lead to apoptosis when they accumulate in excess [26]. Intracellular ROS levels increase dramatically during heat stress, which may affect protein synthesis or cause protein misfolding and damage to the cellular structure, resulting in oxidative stress-induced apoptosis [27]. When cells are stimulated by external stimuli (such as high temperature, hypoxia, and chemical stimulation), it may lead to apoptosis or even death of organisms. At this time, heat shock proteins will be transiently over-synthesized. This defense mechanism is called heat shock response (HRS) [28]. Heat shock protein is a protective protein [29]; however, an excess of heat shock proteins may inhibit cell proliferation, thus causing some detrimental effects, such as the upregulation of DNAJC9 (DnaJ Heat Shock Protein Family (Hsp40) Member C9) expression in gastric adenocarcinoma cells and squamous carcinoma cells of the lungs and the inhibition of cell growth [30]. Most heat shock proteins are heat-induced and are classified according to their molecular weights as small-molecule heat shock proteins (sHSPs), such as Hsp60, Hsp70, Hsp90, and Hsp110. Among them, Hsp27, Hsp70, and Hsp90 play key regulatory roles in heat stress by stabilizing protein conformation or aiding in protein aggregation and dispersion, and thus have been widely studied [31,32].

Based on our previous research, this study established a heat stress model using human intestinal epithelial cells (Caco-2) and investigated the preventive and alleviating actions of vitexin during heat stress to clarify the mechanism of action of vitexin in regulating heat stress, with the aim of alleviating the effects of heat stress.

## 2. Results and Discussion

### 2.1. Vitexin Promotes Caco-2 Cell Proliferation

**Figure 1:**
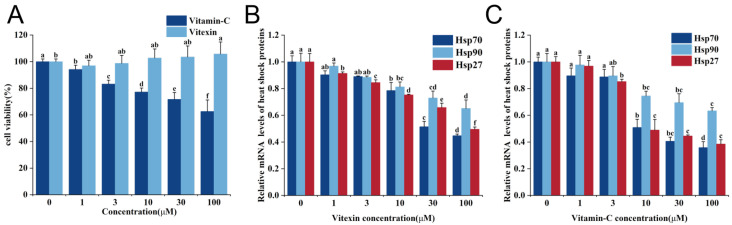
Effect of vitexin and vitamin C on Caco-2 cell viability and expression of heat shock proteins upon heat stress. (**A**) Cell viability detected using CCK-8 after treatment with different concentrations of vitexin and vitamin C for 24 h. (**B**) Effects of different vitexin concentrations on the relative expression levels of *HSPA1A*, *HSP90AB1*, and *HSPB1* in cells during heat stress. (**C**) Effects of different concentrations of vitamin C on the relative expression levels of *HSPA1A*, *HSP90AB1*, and *HSPB1* in cells during heat stress. (Different lowercase letters represent significant differences between different concentrations, *p* < 0.05.)

To determine the effect of vitexin on cell proliferation and its toxicity, we examined the effect of different vitexin concentrations on Caco-2 cells using the CCK-8 assay (Figure 1A). The results showed that vitexin promoted the proliferation of Caco-2 cells in a concentration-dependent manner, and vitexin concentrations of ≤100 μM had no toxic effect on the cells. However, Caco-2 cell survival was negatively correlated with the vitamin C (positive control) concentration compared to vitexin, suggesting that vitamin C has a lower cellular toxicity. Other studies have demonstrated the effects of higher Vc concentrations on the inhibition of cell proliferation [33]. In summary, when the two drugs were administered individually to cells, vitexin had no toxic side effects and was more conducive to cell proliferation.

### 2.2. Effects of Different Vitexin Concentrations on Relative mRNA Expression of HSPA1A, HSP90AB1, and HSPB1

Heat shock proteins are molecular chaperones that help immature proteins to process and fold, but they are not themselves involved in protein expression. Heat shock proteins are synthesized in large quantities when the body is exposed to high temperatures and protect it during normal physiological activities [34]. To select an effective vitexin concentration for heat stress experiments, the relative mRNA expressions of *HSPA1A*, *HSP90AB1*, and *HSPB1* in Caco-2 cells after heat stress were detected using RT-qPCR. The mRNA expression of the three heat shock proteins decreased in a concentration-dependent manner following a treatment with vitexin or vitamin C (Figure 1B,C). Vitexin concentrations of ≥30 μM and vitamin C concentrations of ≥10 μM significantly inhibited the expression of three heat shock proteins.

### 2.3. Vitexin Inhibits Heat-Stress-Induced ROS Production

**Figure 2:**
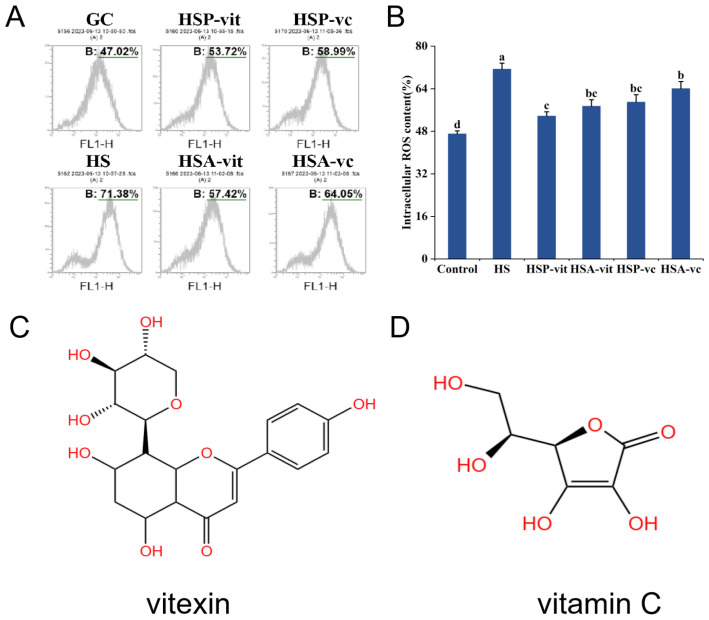
Vitexin in preventing and ameliorating intracellular ROS expression levels during heat stress. (**A**) Detection of intracellular ROS expression using flow cytometry in the prevention and amelioration of heat stress by vitexin. (**B**) Quantification of ROS levels derived from flow cytometry. (**C**) Vitexin’s molecular formula. (**D**) Vitamin C’s molecular formula. (Different lowercase letters represent significant differences between different groups, *p* < 0.05; GC was the control group, HS was the heat stress group, HSP-vit was vitexin for heat stress prevention group, HSA-vit was vitexin for heat stress amelioration group, HSP-vc was the vitamin c for heat stress prevention group, and HSA-vc was the vitamin c for heat stress amelioration group.)

ROS are natural by-products of normal oxygen metabolism, which can play an important role in cellular signal transduction and the regulation of physiological functions in vivo; however, it has been proven that lower ROS concentrations are beneficial to organisms [35]. Excessive ROS can alter cellular redox homeostasis and trigger oxidative stress, thereby inducing apoptosis [36], which can lead to cancer, diabetes, and cardiovascular diseases in severe cases [37,38]. Flow cytometry was used to investigate the cellular ROS levels in the CG, HS, HSP-vit, and HSA-vit group. As shown in Figure 2B, compared to the CG group, the cellular ROS content in the HS group was significantly increased (by 24.36%). Compared with the HS group, the cellular ROS content in the HSP-vit, HSA-vit, HSP-vc, and HSA-vc groups decreased by 17.66%, 13.42%, 12.39%, and 7.33%, respectively. The intracellular ROS content in the HSP-vit and HSA-vit groups decreased by 5.26% and 6.63%, respectively, compared to that in the HSP-vc and HSA-vc groups, respectively, suggesting that vitexin inhibited ROS production more effectively than vitamin C. In addition, the cellular ROS content was reduced by 3.7% in the HSP-vit group compared to that in the HSA-vit group.

There is a close relationship between heat stress and ROS levels. ROS expression in cells increases under heat stress, which affects the balance of the antioxidant system, causes oxidative stress, and leads to cell apoptosis [39]. Natural polyphenols have antioxidant properties, and studies have shown that polyphenol-rich diets can prevent ROS-mediated diseases by inhibiting ROS and attenuating cellular damage, necrosis, and apoptosis [40,41]. When the body is exposed to high temperatures for a long period of time, the level of plasma intestinal fatty acid binding protein (intestinal damage marker) increases, indicating that heat stress damages the intestinal lining, and microorganisms can enter the bloodstream from the intestinal tract, which, if left untreated, can lead to systemic inflammatory reactions [42]. In addition, heat stress was found to alter intestinal permeability by decreasing the expression of tight junction proteins (Occludin and ZO-1) through in vitro experiments, leading to the disruption of the structure and function of the intestinal epithelium [43]. Studies have shown that polyphenols have good antioxidant and anti-inflammatory properties that protect the integrity of the intestinal barrier, and they can also mitigate the effects of heat stress on the body by regulating intestinal flora and pro-inflammatory cytokine expression [44]. Grape seed polyphenols can regulate human intestinal flora and promote the growth of beneficial bacteria, thus improving host health [45]. Berry polyphenols and metabolites can be used as dietary supplements to help the body alleviate heat stress, and it promotes the growth of *Bifidobacterium*, *Lactobacillus*, and *Akkermansia*, and also relieves intestinal inflammation through the modulation of inflammatory factors, and in addition, berry polyphenols have been found to have a cancer-preventive effect on colon cancer [46]. As a natural polyphenol, vitexin can relieve oxidative stress by inhibiting intracellular ROS [47]. In the present study, we treated heat-stressed cells with vitexin and found that it could significantly reduce heat-stress-induced ROS production and that the inhibition of ROS levels was higher in the HSP-vit group than in the HSA-vit group. This may have been attributed to the fact that, in the HSP-vit group, heat-stress-induced ROS was inhibited at the first instance, whereas in the HSA-vit group, ROS was inhibited only when the ROS accumulation was higher. Thus, the effect of ROS inhibition differed between the HSP-vit and HSA-vit groups. ROS generally refer to free and non-free radicals of oxygen origin, including hydrogen peroxide (H_2_O_2_), singlet-linear oxygen (1O_2_), and superoxide anions (O_2_^−^) [48,49]. Studies have shown that hydroxyl groups can react with free radicals and inhibit oxidative reactions. In the antioxidant process, they can undergo a redox reaction with single-linear oxygen, converting it to molecular oxygen and eliminating its activity, thus protecting cellular homeostasis [50]. Under the action of hydroxyl, hydrogen peroxide is broken down into water and oxygen, thereby relieving oxidative stress and protecting cells [51]. In addition, the study confirmed a positive correlation between the number of hydroxyl groups and antioxidant activity [52]. As shown in Figure 2C,D, the structural differences between vitexin and vitamin C are large, and the molecular formula of vitexin has more hydroxyl groups than vitamin C. This may explain why vitexin was more effective in inhibiting ROS. In summary, vitexin inhibited heat-stress-induced ROS production to a certain extent, and the HSP-vit group showed a higher inhibition of ROS production than that of the HSA-vit group.

### 2.4. Vitexin Reduces Heat Shock Protein Expression and Apoptosis Induced by Heat Stress

**Figure 3:**
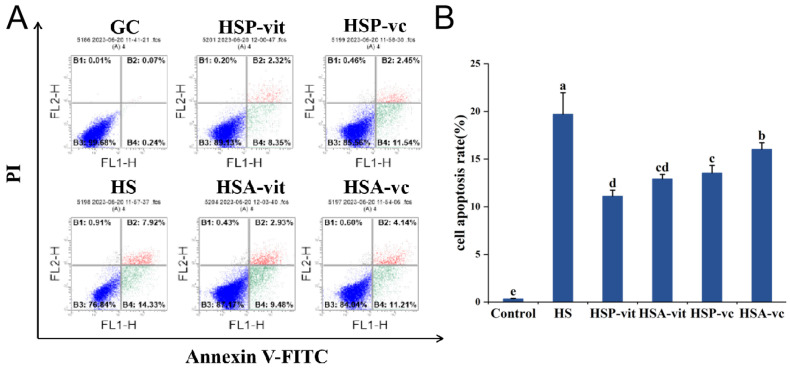
Prevention and amelioration of apoptosis during heat stress by vitexin. (**A**) Detection of apoptosis using flow cytometry in the prevention and amelioration of heat stress by vitexin. (**B**) Quantification of apoptosis derived from flow cytometry. (Different lowercase letters represent significant differences between different groups, *p* < 0.05.)

Apoptosis is the process of programmed cell death. When cells are stimulated by the external environment, they transfer stimulus signals through complex and variable mechanisms to induce apoptosis [53,54]. Therefore, to explore the mechanism of action of vitexin against heat stress injury, flow cytometry was used to detect the effect of vitexin on the rate of apoptosis induced by heat stress (Figure 3B). The apoptosis rate increased significantly (by 21.94%) in the HS group compared to that in the CG group. The apoptosis rate decreased by 11.58% and 9.84% in the HSP-vit and HSA-vit groups, respectively, compared with that in the HS group. The apoptosis rate was reduced by 3.32% and 2.94% in the HSP-vit and HSA-vit groups, respectively, compared to that in the HSP-vc and HSA-vc groups, respectively, suggesting that vitexin protected against heat-stress-induced apoptosis in a slightly better manner than vitamin C. The apoptosis rate in the HSP-vit group was reduced by 1.74% compared to that in the HSA-vit group. Evidently, the prevention or amelioration of heat-stressed cells with vitexin effectively reduced heat-stress-induced apoptosis; however, the mechanism of action is unclear and will be further explored in future studies.

**Figure 4:**
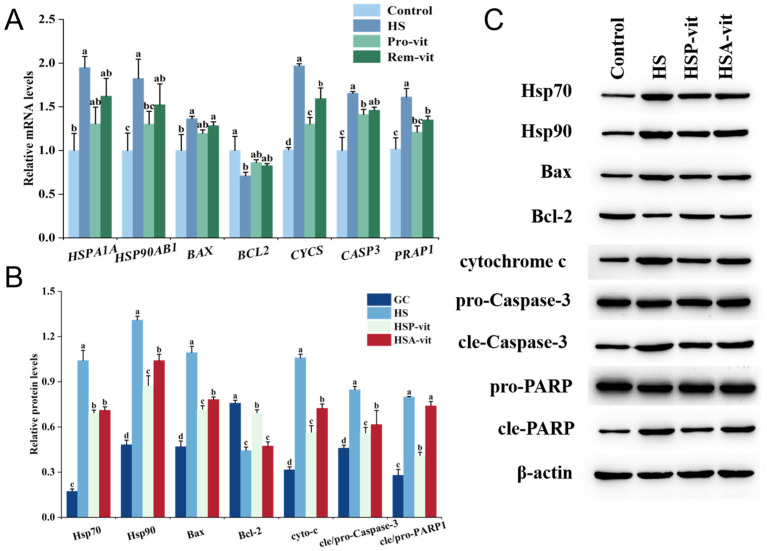
Vitexin reduces heat shock protein expression and heat-stress-induced apoptosis. (**A**) Expression levels of intracellular heat shock proteins and apoptotic proteins were detected using RT-qPCR after the prevention and amelioration of heat stress by vitexin. (**B**) Quantification of Western blot results of heat shock and apoptotic proteins after the prevention and amelioration of heat stress by vitexin with Image-Pro Plus V6.0 software. (**C**) Detection of bands of heat shock proteins and apoptotic proteins after the prevention and amelioration of heat stress by vitexin using Western blot. (Different lowercase letters represent significant differences between different groups, *p* < 0.05.)

The mRNA and protein expression levels of heat shock proteins and key apoptosis factors in the GC, HS, HSP-vit, and HSA-vit groups were detected using RT-qPCR and Western blot. As shown in Figure 4, the results showed that heat stress significantly increased the mRNA expression levels of *HSPA1A* and *HSP90AB1* and the protein expression levels of Hsp70 and Hsp90, whereas the addition of vitexin for heat stress prevention or amelioration reduced the heat-stress-induced elevation in the expression levels of Hsp70 and Hsp90. Heat shock transcription factor 1 (Hsf1) is a master regulator of the heat shock response (HSR), and during heat stress, it drives HSP gene transcription [55]. Varasteh et al. [56] found that the expression of Hsf1 and HSPA1A genes was significantly elevated in Caco-2 cells after heat stress, following the same trend as the results of this study.

We also examined the mRNA and protein expression levels of apoptosis-related factors and found that the mRNA and protein expression levels of the anti-apoptotic protein Bcl-2 were reduced in the HS group compared to those in the GC group. Conversely, the expression levels of *BAX*, *CYCS*, *CASP3*, and *PARP1* mRNA and their proteins (apoptosis proteins Bax, Cytochrome C, cle-Caspase-3, cle-PARP1) were significantly increased, indicating that heat stress affected the expression level of apoptotic proteins by regulating the mRNA expression level of apoptotic proteins to induce apoptosis. Apoptosis mainly involves the intrinsic mitochondrial and extrinsic death receptor pathways, with the mitochondrial pathway being the main transduction pathway [56]. The results of the present study confirmed that heat-stress-induced apoptosis is associated with the caspase-mediated mitochondrial pathway, in which the mRNA expression of *CASP3* and *PARP1* occurs at the gene level, whereas cle-Caspase-3 and cle-PARP1 are expressed at the protein level. Normally, Caspase-3 protein exists in cells as inactive pro-Caspase-3, which is cleaved into cleaved-Caspase-3 active fragments when cells are stimulated by upstream apoptotic signals, whereas the downstream apoptotic signal PARP1 can be cleaved by cleaved-Caspase-3 into cleaved-PARP1 [57,58], which induces apoptosis. Thus, downstream apoptosis is mainly determined at the protein level by the expression levels of cleaved-Caspase-3 and cleaved-PARP1.

Caspase-3 is one of the most important regulators downstream of the caspase cascade, and its activation is largely dependent on the release of cytochrome c [26]. Cytochrome c is an important component of the mitochondrial transport chain [59], and the apoptotic protein Bax of the Bcl-2 family assists small-molecular proteins, such as cytochrome c, to cross the mitochondrial membrane into the cytoplasm to trigger apoptosis, whereas the anti-apoptotic protein Bcl-2 prevents the release of cytochrome c from mitochondria [60]. However, the expression of *BCL2* mRNA and its protein (Bcl-2) in the HSP-vit and HSA-vit groups was increased compared with that in the HS group upon addition of vitexin, while the mRNA expression of *BAX*, *CYCS*, *CASP3*, and *PARP1* and the protein expression of Bax, cytochrome c, cle-Caspase-3, and cle-PARP1 were decreased. These results indicated that vitexin could inhibit the release of cytochrome c from mitochondria by decreasing intracellular Bax expression and increasing Bcl-2 expression, thus decreasing Caspase-3 expression so that PARP1 was not cleaved, effectively alleviating heat-stress-induced apoptosis.

In addition, the mRNA and protein expression of Bcl-2 were increased in the HSP-vit group compared to those in the HSA-vit group, and the mRNA expression of *BAX*, *CYCS*, *CASP3*, and *PARP1* and the protein expression of Bax, cytochrome c, cle-Caspase-3, and cle-PARP were reduced. This indicates that heat-stress-induced apoptosis was inhibited in the HSP-vit group in a slightly better manner than that in the HSA-vit group, which may have been due to the difference in the time point of the action on the heat-stressed cells in the HSP-vit and HSA-vit groups, where the cells were protected immediately when heat stress occurred and its action on the cells lasted for a longer time period. Therefore, the apoptosis rate of cells in the HSP-vit group was lower. In conclusion, vitexin protected against heat-stress-induced apoptosis via the mitochondrial pathway, and apoptosis was inhibited more effectively in the HSP-vit group than that in the HSA-vit group.

### 2.5. Vitexin Inhibited Apoptosis by Regulating Heat Shock Protein Expression

**Figure 5:**
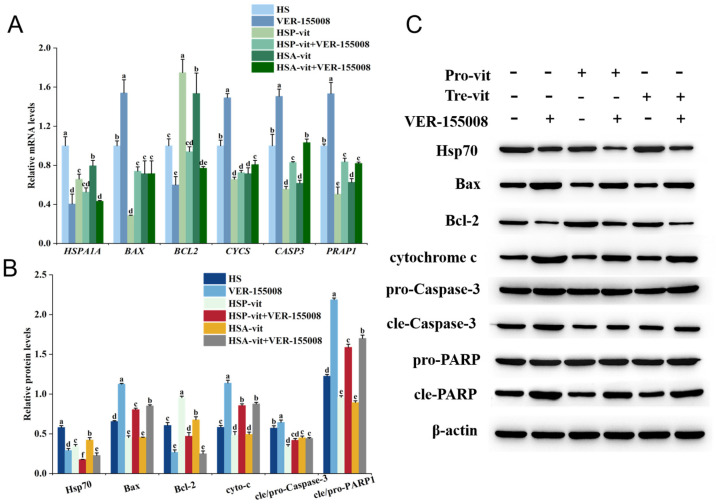
Vitexin reduces intracellular Hsp70 and apoptotic protein expression levels for the prevention and amelioration of heat stress. (**A**) Expression levels of Hsp70 and apoptotic proteins in the prevention and amelioration of heat stress by the addition of VER-155008 inhibitor to vitexin were detected using RT-qPCR. (**B**) Quantification of Western blot results of Hsp70 and apoptotic proteins in the prevention and amelioration of heat stress by the addition of VER-155008 inhibitor to vitexin with Image-Pro Plus V6.0 software. (**C**) Western blot was used to detect bands of Hsp70 and apoptotic proteins in the prevention and amelioration of heat stress by vitexin with the addition of VER-155008 inhibitor. (Different lowercase letters represent significant differences between different groups, *p* < 0.05.)

Heat shock proteins are critical for cell survival and play important roles in apoptosis [61]. Therefore, to further investigate whether vitexin inhibited the rate of apoptosis by modulating heat shock proteins when preventing or ameliorating heat stress, an experiment was conducted to detect the mRNA and protein expression of key factors of apoptosis by adding an Hsp70 inhibitor (VER-155008) and an Hsp90 inhibitor (Tanespimycin). As shown in Figure 5, compared to the HS group, the mRNA expression levels and protein expression levels of *HSPA1A*, *BCL2*, Hsp70, and Bcl-2 were significantly reduced in the VER-155008 group, whereas the mRNA expression of *BAX*, *CYCS*, *CASP3*, and *PARP1*, and the protein expression levels of Bax, cytochrome c, cle-Caspase-3, and cle-PARP1 were significantly increased. Compared to the HSP-vit and HSA-vit groups, the HSP-vit + VER-155008 and HSA-vit + VER-155008 groups showed decreased mRNA and protein expression levels of *HSPA1A*, *BCL2*, Hsp70, and Bcl-2; increased mRNA expression levels of *BAX*, *CYCS*, *CASP3*, and *PARP1*; and increased protein expression levels of Bax, cytochrome c, cle-Caspase-3, and cle-PARP1.

**Figure 6:**
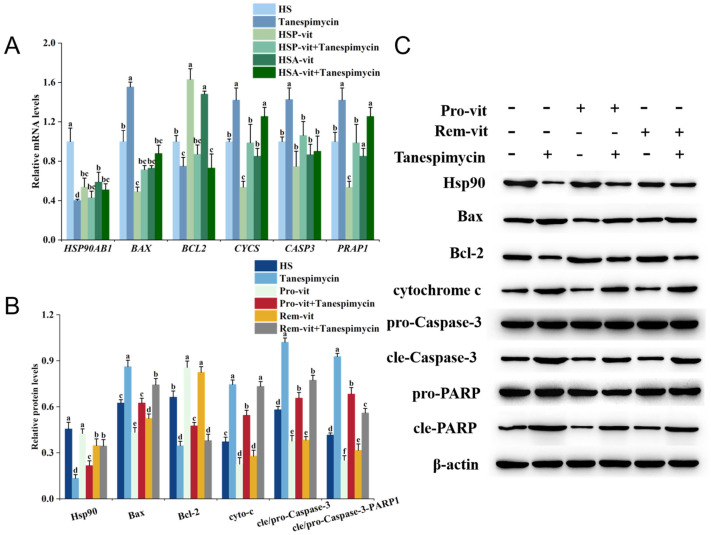
Vitexin reduces intracellular Hsp90 and apoptotic protein expression levels in the prevention and amelioration of heat stress. (**A**) Expression levels of Hsp90 and apoptotic proteins in the prevention and amelioration of heat stress by the addition of Tanespimycin inhibitor to vitexin were detected using RT-qPCR. (**B**) Quantification of Western blot results of Hsp90 and apoptotic proteins in the prevention and amelioration of heat stress by the addition of Tanespimycin inhibitor to vitexin with Image-Pro Plus V6.0 software. (**C**) Western blot was used to detect bands of Hsp90 and apoptotic proteins in the prevention and amelioration of heat stress by vitexin with the addition of Tanespimycin inhibitor. (Different lowercase letters represent significant differences between different groups, *p* < 0.05.)

As shown in Figure 6, the trend of intracellular apoptotic factor expression after the addition of Tanespimycin was consistent with that observed after the addition of VER-155008. Compared to the HS group, the mRNA and protein expression levels of *HSPA1A*, *BCL2*, Hsp90, and Bcl-2 were significantly decreased in the Tanespimycin group, and the mRNA expression levels of *BAX*, *CYCS*, *CASP3*, and *PARP1* and the protein expression levels of Bax, cytochrome c, cle-caspase-3, and cle-PARP1 were significantly increased. Compared to the HSP-vit and HSA-vit groups, the mRNA and protein expression levels of *HSPA1A*, *BCL2*, Hsp90, and Bcl-2 were decreased in the HSP-vit + Tanespimycin and HSA-vit + Tanespimycin groups, and the mRNA expression levels of *BAX*, *CYCS*, *CASP3*, and *PARP1* and the protein expression levels of Bax, cytochrome c, cle-Caspase-3, and cle-PARP1 were increased. However, according to statistical analyses, the elevated mRNA expression level of CASP3 was not significant, indicating that its gene expression was only slightly changed at the mRNA level.

Heat shock proteins are highly conserved proteins that are rapidly synthesized upon external stimulation to maintain cellular homeostasis [62]. In addition, heat shock proteins assist immature protein processing and folding and play a role in regulating apoptosis [63]. However, when effective interventions reduce the effects of external stimuli, heat shock protein expression decreases [64]. The expression levels of Hsp70 and Hsp90 increase rapidly when heat stress occurs, and the heat stress intervention using polyphenols can reduce the expression levels of Hsp70 and Hsp90 [14,65]. However, the role and mechanism of action of Hsp70 and Hsp90 as key regulators of apoptosis in the prevention and amelioration of heat stress by vitexin remain unclear. Therefore, this study was conducted to explore this relationship by adding Hsp70 and Hsp90 inhibitors. The results showed that, compared to the HS group, the expression level of the anti-apoptotic protein Bcl-2 decreased after the addition of VER-155008 and Tanespimycin inhibitors, whereas the expression levels of apoptotic proteins, Bax, cytochrome c, Caspase-3, and PARP1, increased. These results indicate that the low expression of Hsp70 and Hsp90 can further promote apoptosis, whereas the high expression of Hsp70 and Hsp90 can effectively protect against heat-stress-induced apoptosis. However, Hsp70 and Hsp90 expression levels in the HSP-vit and HSA-vit groups were lower than those in the HS group, and apoptosis was reduced. This may have been attributed to the fact that the intervention with vitexin reduced the effects of heat stress on the cells and resulted in the Hsp70 and Hsp90 expression levels being in a more favorable range for protection against heat-stress-induced apoptosis. When vitexin and the inhibitors, VER-155008 or Tanespimycin, acted together on heat-stressed cells, the expression levels of intracellular Bax, cytochrome c, Caspase-3, and PARP1 were lower than those following the addition of the inhibitor VER-155008 or Tanespimycin alone; however, the expression level of Bcl-2 was higher than that when the inhibitor was used alone, which demonstrated that vitexin alleviated heat-stress-induced apoptosis by regulating the expression levels of Hsp70 and Hsp90. In addition, our study demonstrated that vitexin affects the protein expression levels of *HSPA1A* and *HSP90AB1* by downregulating their mRNA transcript levels in cells, thereby alleviating heat stress. In summary, vitexin can alleviate heat-stress-induced apoptosis by regulating the expression of heat shock proteins (Hsp70 and Hsp90) to prevent or ameliorate heat stress.

### 2.6. Vitexin Regulates Heat Shock Protein Expression by Reducing ROS Levels thereby Alleviating Apoptosis

**Figure 7:**
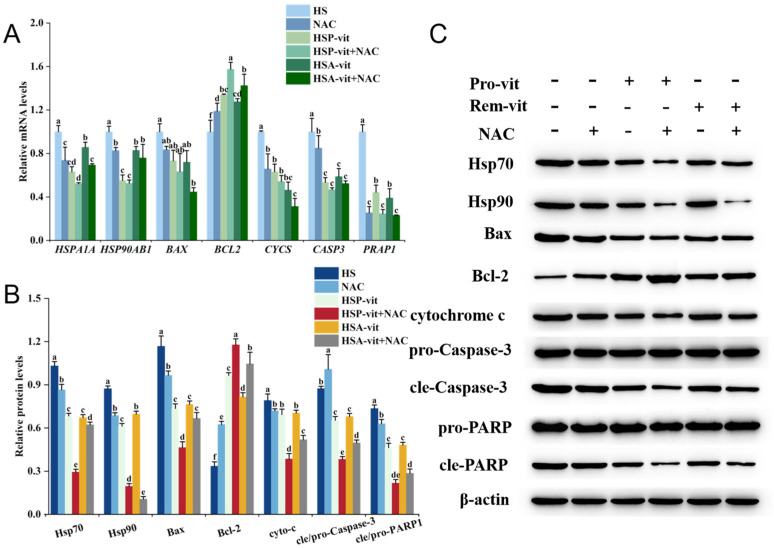
Vitexin reduces intracellular Hsp70, Hsp90, and apoptotic protein expression levels in the prevention and amelioration of heat stress. (**A**) Expression levels of Hsp70, Hsp90, and apoptotic proteins in the prevention and amelioration of heat stress by the addition of NAC inhibitor to vitexin were detected using RT-qPCR. (**B**) Quantification of Western blot results of Hsp70, Hsp90, and apoptotic proteins in the prevention and amelioration of heat stress by the addition of NAC inhibitor to vitexin with Image-Pro Plus V6.0 software. (**C**) Western blot was used to detect bands of Hsp70, Hsp90, and apoptotic proteins in the prevention and amelioration of heat stress by vitexin with the addition of NAC inhibitor. (Different lowercase letters represent significant differences between different groups, *p* < 0.05.)

ROS not only regulate apoptosis but also alter the expression of heat shock proteins [66]. Although vitexin can alleviate heat-stress-induced apoptosis and reduce the expression of intracellular ROS and heat shock proteins, we speculated that it may regulate the expression levels of heat shock proteins and apoptotic proteins by altering ROS levels. Therefore, the mechanism of action was further investigated by adding a ROS inhibitor (NAC). As shown in Figure 7, the mRNA and protein expression levels of *HSPA1A*, *HSP90AB1*, Hsp70, and Hsp90, respectively, were significantly lower in the NAC group than those in the HS group. The mRNA and protein expression levels of *HSPA1A*, *HSP90AB1*, Hsp70, and Hsp90 were reduced in the HSP-vit + NAC and HSA-vit + NAC groups compared to those in the HSP-vit and HSA-vit groups, respectively, suggesting that the downregulation of ROS could reduce intracellular Hsp70 and Hsp90 expression levels. ROS can not only directly alter the structure and activity of heat shock proteins but also indirectly regulate heat shock protein expression through redox signaling pathways [67]. The present study showed that vitexin not only reduced heat-stress-induced ROS production but also inhibited the expression of Hsp70 and Hsp90. Therefore, vitexin may regulate the expression of Hsp70 and Hsp90 by reducing ROS levels.

NAC also altered the expression of apoptotic proteins. As shown in Figure 6, compared to the HS group, the expression levels of *BCL2* mRNA and its protein (Bcl-2) were increased in the NAC group, and the mRNA expression levels of *BAX*, *CYCS*, *CASP3*, and *PARP1* and the protein expression levels of Bax, cytochrome c, cle-Caspase-3, and cle-PARP1 were decreased. Compared with the HSP-vit and HSA-vit groups, the expression levels of *BCL2* mRNA and its protein (Bcl-2) were significantly higher in the HSP-vit + NAC and HSA-vit + NAC groups, whereas the mRNA expression levels of *CYCS*, *CASP3*, and *PARP1* and the protein expression levels of cytochrome c, cle-Caspase-3, and cle-PARP1 were significantly lower, and the mRNA expression levels of *BAX* and the protein expression level of Bax were slightly changed. These results demonstrated that reducing ROS levels may alleviate heat-stress-induced apoptosis. ROS are important factors in the regulation of apoptosis. A high ROS expression can regulate apoptosis through the mitochondrial pathway, and the addition of NAC can effectively inhibit apoptosis [68]. Therefore, based on the above results, we speculate that vitexin is effective in the prevention and amelioration of heat stress by decreasing intracellular ROS levels, thereby inhibiting the expression of heat shock proteins (Hsp70 and Hsp90) and consequently alleviating cell apoptosis.

## 3. Materials and Methods

### 3.1. Materials and Chemicals

Vitexin (Yuanye, Shanghai, China) and vitamin C (Solarbio, Beijing, China) were dissolved in 100% dimethylsulfoxide to prepare a 20 mM stock solution that was stored at −80 °C.

### 3.2. Cell Culture and Compound Treatment

Human intestinal epithelial cells (Caco-2) were purchased from KeyGEN (KeyGEN BioTECH Corp., Ltd., Nanjing, China). Caco-2 cells were cultured in DMEM medium containing 20% fetal bovine serum and 100 U/mL penicillin/streptomycin (Gibco, Thermo Scientific Inc., Waltham, MA, USA) and incubated at 37 °C with 5% CO_2_ saturated humidity. When the cell density reached 70–80%, the cells were digested with 0.25% trypsin (Gibco, Thermo Scientific Inc., Waltham, MA, USA) and passaged.

Referring to the method of Feng et al. [18], cells were treated at 43 °C for 6 h as a heat-stress-modeling condition. The groups were as follows: cells cultured at 37 °C (control group (CG)); cells treated at 43 °C for 6 h followed by recovery at 37 °C for 12 h (heat stress group (HS)); cells pretreated with vitexin (30 μM) for 2 h, followed by treatment with 43 °C for 6 h and recovered at 37 °C for 12 h (vitexin for heat stress prevention group (HSP-vit)); cells treated at 43 °C for 6 h and recovered at 37 °C for 12 h after vitexin (30 μM) addition (vitexin for heat stress amelioration group (HSA-vit)); cells pretreated with vitamin C (10 μM) for 2 h, followed by treatment with 43 °C for 6 h and recovered at 37 °C for 12 h (vitamin C for heat stress prevention group (HSP-vc)); and cells treated at 43 °C for 6 h and recovery at 37 °C for 12 h after vitamin C (10 μM) addition (vitamin C for heat stress amelioration group (HSA-vc)). The inhibitor groups were treated with VER-155008 (Hsp70 inhibitor, MedChemexpress, Shanghai, China), Tanespimycin (Hsp90 inhibitor, MedChemexpress, Shanghai, China), and N-acetyl-L-cysteine (NAC, ROS inhibitor, MedChemexpress, Shanghai, China) for 2 h, and then treated as the HS, HSP-vit, and HSA-vit groups, respectively.

### 3.3. Cell Viability Assay

Cells were seeded into 96-well plates at a density of 1 × 10^4^ cells/well and cultured for 36 h. Vitexin at concentration of 1, 3, 10, 30, or 100 μM was added to the cells for 24 h. Cell Counting Kit-8 (CCK-8) reagent (Biosharp, Hefei, China) was added (10 µL) and incubated for 2 h. Absorbance was measured at 450 nm using an enzyme labeler (SPECTROstar Nano, Berlin, Germany).

### 3.4. Real-Time Quantitative Polymerase Chain Reaction

Cells were seeded into 6-well plates at a density of 1 × 10^5^ cells/well and cultured for 36 h. After the addition of vitexin in concentrations of 1, 3, 10, 30, or 100 μM to the cells for 2 h, the cells were treated at 43 °C for 6 h and recovered at 37 °C for 12 h. After 36 h of cell culture, cells in the inhibitor groups, CG, HS, HSP-vit, and HSA-vit, were treated according to the steps of cell treatment in Section 3.2. Total RNA was extracted from the cells using TRIzol reagent (TransGen, Beijing, China). cDNA was reverse-transcribed from RNA using a PrimeScript RT kit (Takara, Maebashi, Japan). cDNA was subjected to real-time quantitative polymerase chain reaction (RT-qPCR) using a TB Green Premix Ex Taq kit (Takara, Japan) and qTOWER instrument (Analytik Jena AG, Jena, Germany). mRNA expression levels were calculated using the 2^−ΔΔCt^ method. Primer sequences are listed in Table 1.

### 3.5. Measurement of ROS Levels

Cells were seeded into 6-well plates at a density of 1 × 10^5^ cells/well and cultured for 36 h. Cells in the CG, HS, HSP-vit, HSA-vit, HSP-vc, and HSA-vc groups were treated according to the steps for treating cells in Section 3.2. ROS detection was performed using the fluorescent probe DCFH-DA (Beyotime, Shanghai, China). After washing the cells with phosphate-buffered saline (PBS, Biosharp, Guangzhou, China), they were digested with 0.25% trypsin and collected. Then, they were incubated with a DCFH-DA probe in the dark for 30 min at 37 °C. Cells were inverted and mixed every 3–5 min to ensure complete contact between the probe and cells. After washing the cells with a serum-free cell culture medium, intracellular ROS levels were detected using flow cytometry (Sysmex, Norderstedt, Germany).

### 3.6. Cell Apoptosis Analysis

Cells were seeded into 6-well plates at a density of 1 × 10^5^ cells/well and cultured for 36 h. Cells in the CG, HS, HSP-vit, HSA-vit, HSP-vc, and HSA-vc groups were treated according to the steps for treating cells in Section 3.2. Apoptosis was detected using an Annexin V-FITC/PI apoptosis detection kit (Beyotime, Shanghai, China). After washing the cells with PBS, they were digested with 0.25% trypsin and collected, resuspended with 195 μL Annexin V-FITC conjugate, 4 μL Annexin V-FITC, and 8 μL of propidium iodide staining solution. Cells were gently mixed and incubated at room temperature for 20 min. Apoptosis was detected using flow cytometry.

### 3.7. Western Blot Analysis

After 36 h of cell culture, the cells in inhibitor groups, CG, HS, HSP-vit, and HSA-vit, were treated according to the steps of cell treatment in Section 3.2. After washing with PBS, cells were digested with 0.25% trypsin and collected. Cells were lysed in lysis buffer for 30 min. Proteins were separated with electrophoresis on 10–12% sodium dodecyl sulfate-polyacrylamide gel and then transferred to polyvinylidene difluoride membranes, which were blocked in 5% skim milk for 2 h at room temperature. The membranes were washed and incubated with primary antibody (Table 2) for 12 h at 4 °C, and then incubated with murine antibody (Jackson ImmunoResearch Inc., West Grove, PA, USA) for 1 h at room temperature. Proteins were detected using an enhanced chemiluminescence kit (Thermo Fisher Scientific, Waltham, MA, USA) and an Amersham Imager 600 (GE, Fairfield, CT, USA), and analyzed using Image-Pro Plus software.

### 3.8. Data Analysis

The results of the experiments are expressed as the mean ± standard deviation (n ≥ 3). Figures were created using Origin 8.5 (Northampton). Statistical analyses were performed with the one-way analysis of variance and Duncan’s test (*p* < 0.05) using SPSS 25 software.

## 4. Conclusions

**Figure 8:**
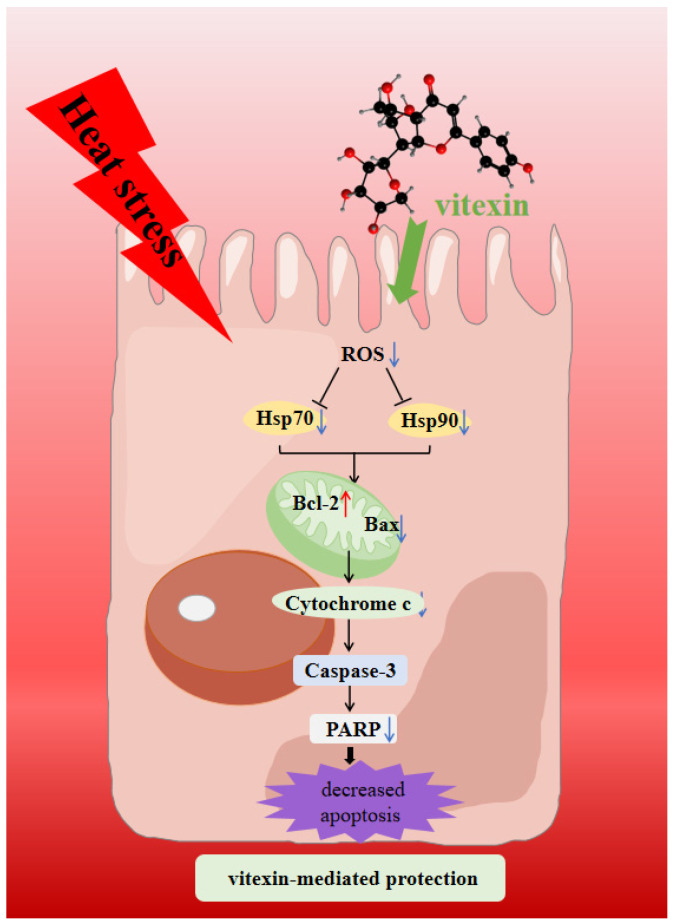
Schematic of the potential mechanism of vitexin-mediated protection against heat-stress-induced apoptosis of Caco-2 cells. (Blue arrows indicate decreased expression and red arrows indicate increased expression).

In this study, we investigated the effects of vitexin on the prevention and amelioration of heat stress and its related mechanisms using in vitro cellular models. It was found that the intervention with vitexin (30 μM) can effectively alleviate heat-stress-induced apoptosis by reducing the intracellular ROS content, thereby inhibiting the expression of intracellular heat shock proteins (Hsp70 and Hsp90) and protecting against the expression of apoptotic proteins (Figure 8). The ROS content and apoptosis expression in the heat stress prevention group were 7.66% and 10.67%, respectively, while the expression in the heat stress amelioration group was slightly higher than that in the prevention group, i.e., 13.96% and 12.41%, respectively. Additionally, the expression of apoptotic proteins was lower in the vitexin-preventing heat stress group than in the vitexin-ameliorating heat stress group. In conclusion, vitexin effectively prevented and alleviated heat-stress-induced apoptosis, and its preventive effect on heat stress was more effective than its ameliorative effect. Vitexin has potential applications in preventing and alleviating heat stress, and this study provides a theoretical basis for the regulation of heat stress by vitexin. In future studies, we will investigate the mechanism by which vitexin regulates heat-stress-related signaling pathways to further elucidate the mechanism of action of vitexin in alleviating heat stress.

## Figures and Tables

**Table 1:** Primer sequence list.

Gene	Sequences 5′–3′(Forward/Reverse)	Annealing Temperature (°C)	Product Size (bp)
*HSPA1A*	TTGAAGAGCAACAGCAGCAG GCTGATTCTCGATTGGCAGG	60	248
*HSP90AB1*	GAAACTGCGCTCCTGTCTTC GAAACTGCGCTCCTGTCTTC	60	239
*HSPB1*	GATGGCGTGGTGGAGATCA GGTGACTGGGATGGTGATCT	60	150
*BAX*	AAGAAGCTGAGCGAGTGTCT GTTCTGATCAGTTCCGGCAC	60	181
*BCL2*	GGATAACGGAGGCTGGGATG TGACTTCACTTGTGGCCCAG	60	156
*CYCS*	ACAGAAACCAGGCAGCCTTT CAGGGACTGTGCTCTGGAAG	60	123
*CASP3*	GGCGGTTGTAGAAGAGTTTCG ACACAGCCACAGGTATGAGC	60	53
*PRAP1*	AGCGTGTTTCTAGGTCGTGG CATCAAACATGGGCGACTGC	60	194
*ACTB*	CCCTGGAGAAGAGCTACGAG CGTACAGGTCTTTGCGGATG	60	240

**Table 2:** Primary antibody information.

Name of Primary Antibody	Species of Origin	Producer	Article Number	Dilution Rate
HSP90	Rabbit	Mitaka, Wuhan, China	13171-1-AP	1:5000
HSP70	Rabbit	Affinity, Changzhou, China	AF5466	1:1000
Bax	Rabbit	CAT, Danvers, MA, USA	2772	1:1000
Bcl-2	Rabbit	Mitaka, Wuhan, China	26593-1-AP	1:2000
cytochrome c	Rabbit	Mitaka, Wuhan, China	10993-1-AP	1:3000
pro-PARP1	Rabbit	Huaan biology, Hangzhou, China	ET1608-56	1:2000
cle-PARP1	Rabbit	Affinity, Changzhou, China	AF7023	1:1000
pro-caspase3	Rabbit	Abcam, Shanghai, China	ab32150	1:1000
cle-caspase3	Rabbit	Affinity, Changzhou, China	AF7022	1:1000
β-actin	Rabbit	Abclonal, Wuhan, China	AC026	1:50,000

## Data Availability

The data presented in this study are available on request from the corresponding author.

## References

[1] Hu Y., Lin L., Liu K., Liu E., Han S., Gong Z., Xiao W. (2023). L-Theanine alleviates heat stress-induced impairment of immune function by regulating the p38 MAPK signalling pathway in mice. Food Funct..

[2] Bagath M., Krishnan G., Devaraj C., Rashamol V.P., Pragna P., Lees A.M., Sejian V. (2019). The impact of heat stress on the immune system in dairy cattle: A review. Res. Vet. Sci..

[3] Périard J.D., Eijsvogels T.M.H., Daanen H.A.M. (2021). Exercise under heat stress: Thermoregulation, hydration, performance implications, and mitigation strategies. Physiol. Rev..

[4] Parkes B., Buzan J.R., Huber M. (2022). Heat stress in Africa under high intensity climate change. Int. J. Biometeorol..

[5] Khan A.A. (2019). Heat related illnesses. Review of an ongoing challenge. Saudi Med. J..

[6] Salata F., Golasi I., Petitti D., de Lieto Vollaro E., Coppi M., de Lieto Vollaro A. (2017). Relating microclimate, human thermal comfort and health during heat waves: An analysis of heat island mitigation strategies through a case study in an urban outdoor environment. Sustain. Cities Soc..

[7] Kovats R.S., Hajat S. (2008). Heat stress and public health: A critical review. Annu. Rev. Public Health.

[8] Sorensen C., Hess J. (2022). Treatment and Prevention of Heat-Related Illness. N. Engl. J. Med..

[9] Hu R., He Y., Arowolo M.A., Wu S., He J. (2019). Polyphenols as Potential Attenuators of Heat Stress in Poultry Production. Antioxidants.

[10] Wu J., Ibtisham F., Niu Y.F., Wang Z., Li G.H., Zhao Y., Nawab A., Xiao M., An L. (2019). Curcumin inhibits heat-induced oxidative stress by activating the MAPK-Nrf2/ARE signaling pathway in chicken fibroblasts cells. J. Therm. Biol..

[11] He S., Guo Y., Zhao J., Xu X., Song J., Wang N., Liu Q. (2019). Ferulic acid protects against heat stress-induced intestinal epithelial barrier dysfunction in IEC-6 cells via the PI3K/Akt-mediated Nrf2/HO-1 signaling pathway. Int. J. Hyperth..

[12] Putics A., Vegh E.M., Csermely P., Sőti C. (2008). Resveratrol induces the heat-shock response and protects human cells from severe heat stress. Antioxid. Redox Signal..

[13] Yin B., Lian R., Li Z., Liu Y., Yang S., Huang Z., Zhao Z., Li Y., Sun C., Lin S. (2021). Tea Polyphenols Enhanced the Antioxidant Capacity and Induced Hsps to Relieve Heat Stress Injury. Oxidative Med. Cell. Longev..

[14] Liu L., Fu C., Yan M., Xie H., Li S., Yu Q., He S., He J. (2016). Resveratrol modulates intestinal morphology and HSP70/90, NF-κB and EGF expression in the jejunal mucosa of black-boned chickens on exposure to circular heat stress. Food Funct..

[15] Ding C., Shen H., Tian Z., Kang M., Ma J., He Q., Wang J., Zhang Y., Deng Y., Wang D. (2021). Protective effect of hawthorn vitexin on the ethanol-injured DNA of BRL-3A hepatocytes. Medicine.

[16] Zhang D., Ning T., Wang H. (2022). Vitexin alleviates inflammation and enhances apoptosis through the regulation of the JAK/STAT/SOCS signaling pathway in the arthritis rat model. J. Biochem. Mol. Toxicol..

[17] Bhardwaj M., Paul S., Jakhar R., Kang S.C. (2015). Potential role of vitexin in alleviating heat stress-induced cytotoxicity: Regulatory effect of Hsp90 on ER stress-mediated autophagy. Life Sci..

[18] Feng Y., Fan X., Suo D., Zhang S., Ma Y., Wang H., Guan X., Yang H., Wang C. (2022). Screening of heat stress-regulating active fractions in mung beans. Front. Nutr..

[19] Senthamilan S., Aggarwal A., Grewal S., Rani S., Vats P., Pal P., Jaswal S., Arya A., Alhussien M.N. (2023). Pre-treatment but not co-treatment with vitexin alleviates hyperthermia induced oxidative stress and inflammation in buffalo mammary epithelial cells. J. Reprod. Immunol..

[20] Cheng K., Song Z., Li S., Yan E., Zhang H., Zhang L., Wang C., Wang T. (2019). Effects of resveratrol on intestinal oxidative status and inflammation in heat-stressed rats. J. Therm. Biol..

[21] Dou M., Zhang Y., Shao Q., Zhu J., Wang X., Zhang C., Li Y. (2021). L-arginine reduces injury from heat stress to bovine intestinal epithelial cells by improving antioxidant and inflammatory response. Anim. Biotechnol..

[22] Swank G.M., Lu Q., Xu D.Z., Michalsky M., Deitch E.A. (1998). Effect of acute-phase and heat-shock stress on apoptosis in intestinal epithelial cells (Caco-2). Crit. Care Med..

[23] Pardo Z., Seiquer I. (2021). Supplemental Zinc exerts a positive effect against the heat stress damage in intestinal epithelial cells: Assays in a Caco-2 model. J. Funct. Foods.

[24] Hengartner M.O. (2000). The biochemistry of apoptosis. Nature.

[25] White E. (1996). Life, death, and the pursuit of apoptosis. Genes Dev..

[26] Sheng Y.N., Luo Y.H., Liu S.B., Xu W.T., Zhang Y., Zhang T., Xue H., Zuo W.B., Li Y.N., Wang C.Y. (2020). Zeaxanthin Induces Apoptosis via ROS-Regulated MAPK and AKT Signaling Pathway in Human Gastric Cancer Cells. OncoTargets Ther..

[27] Liu Y., Wang Z., Xie W., Gu Z., Xu Q., Su L. (2017). Oxidative stress regulates mitogen-activated protein kinases and c-Jun activation involved in heat stress and lipopolysaccharide-induced intestinal epithelial cell apoptosis. Mol. Med. Rep..

[28] Kim S., Kim Y., Suh D.H., Lee C.H., Yoo S.M., Lee S.Y., Yoon S.H. (2020). Heat-responsive and time-resolved transcriptome and metabolome analyses of Escherichia coli uncover thermo-tolerant mechanisms. Sci. Rep..

[29] Dukay B., Csoboz B., Tóth M.E. (2019). Heat-Shock Proteins in Neuroinflammation. Front. Pharmacol..

[30] Zhang Z., Jing J., Ye Y., Chen Z., Jing Y., Li S., Hong W., Ruan H., Liu Y., Hu Q. (2020). Characterization of the dual functional effects of heat shock proteins (HSPs) in cancer hallmarks to aid development of HSP inhibitors. Genome Med..

[31] Zhang X., Yu W. (2022). Heat shock proteins and viral infection. Front. Immunol..

[32] Bastaki N.K., Albarjas T.A., Almoosa F.A., Al-Adsani A.M. (2023). Chronic heat stress induces the expression of HSP genes in the retina of chickens (Gallus gallus). Front. Genet..

[33] Qiu J., Wu R., Long Y., Peng L., Yang T., Zhang B., Shi X., Liu J., Zhang X. (2022). Role of Fe, Transferrin and Transferrin Receptor in Anti-Tumor Effect of Vitamin C. Cancers.

[34] Yang H., Chowdhury V.S., Han G., Zhang R., Furuse M. (2019). Flavangenol regulates gene expression of HSPs, anti-apoptotic and anti-oxidative factors to protect primary chick brain cells exposed to high temperature. J. Therm. Biol..

[35] Costa M., Sezgin-Bayindir Z., Losada-Barreiro S., Paiva-Martins F., Saso L., Bravo-Díaz C. (2021). Polyphenols as Antioxidants for Extending Food Shelf-Life and in the Prevention of Health Diseases: Encapsulation and Interfacial Phenomena. Biomedicines.

[36] Liu L., Liu Y., Cheng X., Qiao X. (2021). The Alleviative Effects of Quercetin on Cadmium-Induced Necroptosis via Inhibition ROS/iNOS/NF-κB Pathway in the Chicken Brain. Biol. Trace Elem. Res..

[37] Chen W., Li D. (2020). Reactive Oxygen Species (ROS)-Responsive Nanomedicine for Solving Ischemia-Reperfusion Injury. Front. Chem..

[38] Zhang Y., Xi X., Mei Y., Zhao X., Zhou L., Ma M., Liu S., Zha X., Yang Y. (2019). High-glucose induces retinal pigment epithelium mitochondrial pathways of apoptosis and inhibits mitophagy by regulating ROS/PINK1/Parkin signal pathway. Biomed. Pharmacother..

[39] Wang R., Shi Z., Li J., Tang D., Qin S., Guo Y. (2022). Protective Effect of Manganese on Apoptosis and Mitochondrial Function of Heat-Stressed Primary Chick Embryonic Myocardial Cells. Biol. Trace Elem. Res..

[40] Wu X., Kong W., Qi X., Wang S., Chen Y., Zhao Z., Wang W., Lin X., Lai J., Yu Z. (2019). Icariin induces apoptosis of human lung adenocarcinoma cells by activating the mitochondrial apoptotic pathway. Life Sci..

[41] Lončar A., Negrojević L., Dimitrić-Marković J., Dimić D. (2021). The reactivity of neurotransmitters and their metabolites towards various nitrogen-centered radicals: Experimental, theoretical, and biotoxicity evaluation. Comput. Biol. Chem..

[42] Foster J., Mckenna Z.J., Atkins W.C., Jarrard C.P., Crandall C.G. (2023). Aging Increases Enterocyte Damage during a 3-Hour Exposure to Very Hot and Dry Heat: A Preliminary Study. Biology.

[43] Peng M., Yi W., Murong M., Peng N., Tong H., Jiang M., Jin D., Peng S., Liang W., Quan J. (2023). Akkermansia muciniphila improves heat stress-impaired intestinal barrier function by modulating HSP27 in Caco-2 cells. Microb. Pathog..

[44] King M.A., Rollo I., Baker L.B. (2021). Nutritional considerations to counteract gastrointestinal permeability during exertional heat stress. J. Appl. Physiol..

[45] Zhou L., Wang W., Huang J., Ding Y., Pan Z., Zhao Y., Zhang R., Hu B., Zeng X. (2016). In vitro extraction and fermentation of polyphenols from grape seeds (*Vitis vinifera*) by human intestinal microbiota. Food Funct..

[46] Lavefve L., Howard L.R., Carbonero F. (2020). Berry polyphenols metabolism and impact on human gut microbiota and health. Food Funct..

[47] Li S., Liang T., Zhang Y., Huang K., Yang S., Lv H., Chen Y., Zhang C., Guan X. (2021). Vitexin alleviates high-fat diet induced brain oxidative stress and inflammation via anti-oxidant, anti-inflammatory and gut microbiota modulating properties. Free Radic. Biol. Med..

[48] Borisov V.B., Siletsky S.A., Nastasi M.R., Forte E. (2021). ROS Defense Systems and Terminal Oxidases in Bacteria. Antioxidants.

[49] Dimić D., Milenković D., Marković J.D., Marković Z. (2017). Antiradical activity of catecholamines and metabolites of dopamine: Theoretical and experimental study. Phys. Chem. Chem. Phys..

[50] Tasaka T., Matsumoto T., Nagashima U., Nagaoka S.I. (2023). Potential energy curve for singlet-oxygen quenching reaction by vitamin E. J. Photochem. Photobiol. A Chem..

[51] Kalyanaraman B., Hardy M., Podsiadly R., Cheng G., Zielonka J. (2017). Recent developments in detection of superoxide radical anion and hydrogen peroxide: Opportunities, challenges, and implications in redox signaling. Arch. Biochem. Biophys..

[52] Wang Y., Liu C., Tang S., Tian J., Wang Y., Yang Y. (2023). Thermodynamics, kinetics and structure-activity relationship of hydroxyanthraquinones scavenging free radicals. Food Biosci..

[53] Chu Q., Gu X., Zheng Q., Wang J., Zhu H. (2021). Mitochondrial Mechanisms of Apoptosis and Necroptosis in Liver Diseases. Anal. Cell. Pathol..

[54] Deng Y., Li X., Li X., Zheng Z., Huang W., Chen L., Tong Q., Ming Y. (2018). Corilagin induces the apoptosis of hepatocellular carcinoma cells through the mitochondrial apoptotic and death receptor pathways. Oncol. Rep..

[55] Zhang H., Shao S., Zeng Y., Wang X., Qin Y., Ren Q., Xiang S., Wang Y., Xiao J., Sun Y. (2022). Reversible phase separation of HSF1 is required for an acute transcriptional response during heat shock. Nat. Cell Biol..

[56] Gogvadze V., Orrenius S., Zhivotovsky B. (2006). Multiple pathways of cytochrome c release from mitochondria in apoptosis. Biochim. Biophys. Acta.

[57] Kim S.Y., Yi H.K., Yun B.S., Lee D.Y., Hwang P.H., Park H.R., Kim M.S. (2020). The extract of the immature fruit of Poncirus trifoliata induces apoptosis in colorectal cancer cells via mitochondrial autophagy. Food Sci. Hum. Wellness.

[58] El-Zarei M.F., Alseaf A.M., Alhaidary A.A., Mousa E.F., Okab A.B., Samara E.M., Abdoun K.A. (2019). Short-term heat shock proteins 70 and 90 mRNA expression profile and its relation to thermo-physiological parameters in goats exposed to heat stress. Int. J. Biometeorol..

[59] Zhao H., Yenari M.A., Cheng D., Sapolsky R.M., Steinberg G.K. (2003). Bcl-2 overexpression protects against neuron loss within the ischemic margin following experimental stroke and inhibits cytochrome c translocation and caspase-3 activity. J. Neurochem..

[60] Ahmadian E., Eftekhari A., Babaei H., Nayebi A.M., Eghbal M.A. (2017). Anti-Cancer Effects of Citalopram on Hepatocellular Carcinoma Cells Occur via Cytochrome C Release and the Activation of NF-kB. Anticancer Agents Med. Chem..

[61] Kennedy D., Jäger R., Mosser D.D., Samali A. (2014). Regulation of apoptosis by heat shock proteins. IUBMB Life.

[62] Pawar S.S., Kurade N.P., Bhendarkar M.P., Bhosale S.V., Nirmale A.V., Kochewad S.A. (2023). Modulation of heat shock protein 70 (HSP70) gene expression ex vivo in response to heat stress in chicken. Anim. Biotechnol..

[63] Shim Y.Y., Tse T.J., Saini A.K., Kim Y.J., Reaney M.J. (2022). Uptake of Flaxseed Dietary Linusorbs Modulates Regulatory Genes Including Induction of Heat Shock Proteins and Apoptosis. Foods.

[64] Yang H., Chowdhury V.S., Bahry M.A., Tran P.V., Do P.H., Han G., Zhang R., Tagashira H., Tsubata M., Furuse M. (2016). Chronic oral administration of pine bark extract (flavangenol) attenuates brain and liver mRNA expressions of HSPs in heat-exposed chicks. J. Therm. Biol..

[65] Yang J.C., Myung S.C., Kim W., Lee C.S. (2012). 18β-glycyrrhetinic acid potentiates Hsp90 inhibition-induced apoptosis in human epithelial ovarian carcinoma cells via activation of death receptor and mitochondrial pathway. Mol. Cell. Biochem..

[66] Abdelnour S.A., Swelum A.A., Abd El-Hack M.E., Khafaga A.F., Taha A.E., Abdo M. (2020). Cellular and functional adaptation to thermal stress in ovarian granulosa cells in mammals. J. Therm. Biol..

[67] Zhang H., Gong W., Wu S., Perrett S. (2022). Hsp70 in Redox Homeostasis. Cells.

[68] Zhang T., Li S.M., Li Y.N., Cao J.L., Xue H., Wang C., Jin C.H. (2022). Atractylodin Induces Apoptosis and Inhibits the Migration of A549 Lung Cancer Cells by Regulating ROS-Mediated Signaling Pathways. Molecules.

